# Postnatal levels of glycated albumin and glycated hemoglobin A1c in mothers of large-for-gestational-age newborns

**DOI:** 10.3389/fped.2024.1439876

**Published:** 2024-11-12

**Authors:** Mojca Železnik, Alenka Trampuš Bakija, Darja Paro-Panjan, Aneta Soltirovska-Šalamon

**Affiliations:** ^1^ Department of Neonatology, University Children's Hospital, University Medical Centre Ljubljana, Ljubljana, Slovenia; ^2^ Clinical Institute of Special Laboratory Diagnostics, University Children's Hospital, University Medical Centre Ljubljana, Ljubljana, Slovenia; ^3^ Department of Pediatrics, Faculty of Medicine, University of Ljubljana, Ljubljana, Slovenia

**Keywords:** gestational diabetes mellitus, glycated albumin, glycated hemoglobin, large for gestational age, newborn

## Abstract

**Background:**

Gestational diabetes mellitus (GDM) is an important cause of macrosomia. The value of glycated albumin (GlyA) has been demonstrated to be a useful marker of glycemic control in pregnancy and a predictor of adverse perinatal outcomes. The aim of this study was to investigate the relationship between the postnatal levels of GlyA and glycated hemoglobin A1c (HbA1c) regarding the prenatal diagnosis of GDM in mothers of large-for-gestational-age (LGA) newborns.

**Methods:**

The study included mothers and their LGA newborns born between July 2017 and September 2019. The mothers were grouped according to the prenatal diagnosis of GDM, and measurements of GlyA and HbA1c levels in their serum were performed on the first day after delivery of a LGA newborn.

**Results:**

A total of 61 LGA newborns and their mothers were enrolled in the study. The median GlyA level was higher, at 16.4% (81.0 µmol/L), whereas the HbA1c level was lower in the group without a prenatal diagnosis of GDM; the differences between groups regarding the GlyA and HbA1c levels were not significant (*p* > 0.05). The postnatal level of maternal GlyA was positively correlated with birth weight (*β* = 0.022, *p* = 0.007), but no correlation with the presence of other adverse perinatal outcomes was found.

**Conclusion:**

Mothers of LGA newborns who were not diagnosed with GDM during pregnancy had higher median levels of GlyA and lower HbA1c levels than mothers with prenatal diagnosis of GDM. Values of GlyA in mothers were positively correlated with the birth weight of their newborns but no correlation with other adverse perinatal outcomes was found. Our results indicate the potential value of GlyA for screening of GDM in the last trimester of pregnancy.

## 1 Introduction

Anthropometric measurements at birth are an important parameter for predicting the health status of newborns, and are closely related to postnatal morbidity and complications during childbirth, as well as to long-term consequences (1–3). Large-for-gestational-age (LGA) newborns are defined as having birth weight and/or birth length above the 90th percentile for gestational age and sex (4). They are at increased risk for adverse perinatal outcomes, including hypoglycemia, polycythemia, hyperbilirubinemia, respiratory distress and the need for mechanical ventilation, traumatic delivery, congenital malformations, and neonatal mortality (5, 6). In addition, studies have shown that being born LGA is associated with an increased risk of morbidity later in life, which underscores the significance of anthropometric measures as alternative clinical parameters for *in utero* programming (7–10). Etiologically, apart from maternal obesity, gestational diabetes mellitus (GDM) is the most important cause of macrosomia (11). It is defined as impaired glucose tolerance of variable degrees, with onset or being first recognized during pregnancy (12). In developing countries, the prevalence of GDM has increased significantly in recent years and is expected to increase further in the future (12, 13). Studies have shown that 15%–45% of newborns born to mothers with GDM can be macrosomic, a rate that is three times higher compared to those born to normoglycemic mothers (14). According to the World Health Organization and International Association of Diabetes and Pregnancy Study Groups, GDM should be diagnosed when the fasting plasma glucose level is ≥5.1 mmol/L in the first trimester of pregnancy, or when it is diagnosed between the 24th and 28th week of pregnancy following a 75-g oral glucose load if the 1-h plasma glucose level is ≥10.0 mmol/L or the 2-h plasma glucose level is ≥8.5 mmol/L (15, 16).

Glycated hemoglobin A1c (HbA1c) is one of the most commonly used biomarkers for evaluating long-term glycemic control, and reflects changes in plasma glucose values over the preceding 2–3 months (13, 17, 18). One of its limitations is the influence of external factors such as erythrocyte lifespan and other factors known to affect red blood cell survival, such as hyperglycemia (19, 20). Indices revealed that HbA1c levels are insufficient for monitoring glycemic control in pregnancy, and it has been reported that glycated albumin (GlyA) is a useful marker of glycemic control (21–23). Its value as an alternative glycemic marker during pregnancy has been examined in several studies (6, 18, 24–26). GlyA is not affected by hemoglobin metabolism or iron-deficiency anemia, and it reflects the status of blood glucose more rapidly than HbA1c; this makes it more sensitive indicator of intermediate-term glycemia, capturing fluctuations in blood glucose over the past 2–3 weeks (17, 20, 24, 27).

Hiramatsu et al. defined the reference intervals of GlyA in healthy pregnant Japanese women for each trimester (18). Furthermore, the cut-off values of GlyA levels in women with GDM for predicting adverse perinatal outcomes in their newborns were reported to be 13.6%–14.7% (28). Japanese authors also found a positive correlation between GlyA levels in diabetic mothers and the number of adverse perinatal outcomes in their newborns (28). Conversely, Dong et al. found a decreasing trend of GlyA levels throughout the pregnancy, and concluded that the GlyA level has limited importance for the diagnosis of GDM in the late second trimester and in predicting birth outcomes (29).

To further elucidate the relationship between GlyA levels, prenatal diagnosis of GDM, and the most common complication of GDM (delivery of an LGA newborn), we aimed to explore postnatal values of GlyA in the serum of mothers who delivered LGA newborns. Assuming that the level of GlyA reflects intermediate-term glycemia in pregnancy, we hypothesized that (i) mothers of LGA newborns who were not diagnosed with GDM during pregnancy using standard procedures had higher values of GlyA and (ii) the value of maternal GlyA after delivery is positively correlated with birth weight and other adverse perinatal outcomes.

## 2 Materials and methods

### 2.1 Study population

A cross-sectional study was conducted at the Department of Neonatology, University Children's Hospital, University Medical Centre Ljubljana (approximately 500 referrals per year). The study included mothers and their newborns born between July 2017 and September 2019. Newborns born at ≥36 weeks of gestation as LGA [birth weight, length, or head circumference above the 90th percentile for gestational age and sex according to the growth curve for Slovenian singletons in 2000 (30)] and their mothers were studied. Birth weight was recorded to the nearest 10 g. Exclusion criteria were: gestational age <36 weeks, perinatal hypoxia (Apgar score <5 at 10 min), the need for mechanical ventilation, sepsis (clinical characteristics and positive blood culture) and thyroid dysfunction. Prenatal, perinatal and postnatal clinical and laboratory data were collected.

Adverse perinatal outcomes in newborns were defined as follows: Hypoglycemia was defined as a blood glucose threshold of <2.61 mmol/L (47 mg/dl) (31). A venous hematocrit level >65% or a hemoglobin concentration >220 g/L (≥22 g/dl) was defined as polycythemia (32). Structural or functional heart abnormalities were defined when detected by electrocardiogram or echocardiogram due to clinical suspicion (33). Hyperbilirubinemia was defined if the newborn was clinically jaundiced and required phototherapy (34). Newborns showing one or more clinical signs of respiratory distress for >2 h and requiring oxygen support were considered to have respiratory distress. Neurological examination was performed and graded using the Amiel–Tison Neurologic Evaluation; mildly abnormal neurological signs were defined as abnormalities in muscle tone and excitability (35). Birth trauma was defined as skin damage, extracranial injury of the head, facial or brachial nerve palsy, a fractured bone, or adrenal hemorrhage. The structural abnormality of a single organ, with the exception of heart abnormalities, was defined as anomalies. The number of adverse perinatal outcomes was defined as the sum of all previously mentioned categories.

Mothers of LGA newborns were grouped according to the diagnosis of GDM during pregnancy. The diagnosis followed the Slovenian national guidelines: fasting plasma glucose level ≥5.1 mmol/L in the first trimester of pregnancy, or 1-h plasma glucose level ≥10.0 mmol/L, or the 2-h plasma glucose ≥8.5 mmol/L after a 75-g oral glucose tolerance test (OGTT) performed between the 24th and 28th week of pregnancy (36). Mothers diagnosed with GDM self-monitored their blood glucose levels, preprandial and postprandial, to achieve target fasting blood glucose levels between 3.5 and 5.3 mmol/L and 90 min after meals ≤6.6 mmol/L. HbA1c values were monitored every 4–8 weeks. Mothers with prepartum body mass index >25 kg/m^2^, multiple pregnancies, conception after infertility treatment, serum albumin below 25 g/L, abnormal renal function, dyslipidemia, hyperthyroidism, liver cirrhosis, and hematological disorders were excluded from the study. The following data were collected: maternal age, type of diabetes and treatment during pregnancy, blood pressure during pregnancy, and smoking status.

Measurement of GlyA and HbA1c levels in the mother’s serum was performed within the first day after delivery of the LGA newborn.

### 2.2 Laboratory methods

The GlyA measurements were performed at the Clinical Institute of Special Laboratory Diagnostics, University Children's Hospital, University Medical Centre Ljubljana. GlyA results were calculated as the ratio of GlyA to albumin. Albumin was assayed on an automated biochemical analyzer (Olympus AU400; Beckman Coulter, CA, USA) using the bromocresol green method (albumin cat. no. OSR 6102, Beckman Coulter). GlyA was measured using a manual competitive inhibition enzyme-linked immunosorbent assay (Human GlyA ELISA kit; cat. no. EKC33864, Biomatik, Canada). Briefly, samples/standards and horseradish peroxidase conjugated antibodies specific for GlyA were added to the microtiter plate wells. After the incubation, wells were washed and tetramethylbenzidine substrate solution was added. The color development is stopped and the intensity of color is measured at 450 nm using microplate reader.

### 2.3 Statistical analysis

The sample size was calculated to have sufficient statistical power to detect the diagnostic effect of GlyA determination in mothers who delivered LGA newborns. Data analyses were performed using IBM SPSS Statistics (version 22; IBM Corp., Armonk, NY, USA). Means and standard deviations (SDs) or medians and interquartile ranges (IQRs) were calculated as appropriate. For normally distributed variables, the independent samples *t*-test was used to compare means. A nonparametric Wilcoxon signed-rank test was used to test the difference in the mean rank between the two groups. For the correlation between two nominal variables, the Pearson chi-square test was used, corrected for a 2 × 2 table. Multiple linear regression analysis was used to examine the relationship between GlyA levels and explanatory variables. Statistical significance was set at *p* < 0.05.

### 2.4 Statement of ethics

Research complied with all relevant national regulations, institutional policies and is in accordance with the tenets of the Declaration of Helsinki and has been approved by the National Medical Ethics Committee (approval no. 0120-582/2017/5). Written informed consent was obtained from all the individuals included in this study.

## 3 Results

### 3.1 Clinical characteristics of LGA newborns

Our study population consisted of 61 LGA newborns, with 38 (62%) boys. Their gestational age was 39 ± 1 weeks, and the mean birth weight was 4,268 ± 347.1 g (range 3,650–5,420 g). Adverse perinatal outcomes were found in 31 (51%) newborns. Twelve (20%) had abnormal neurological signs (three presenting abnormalities in muscle tone and others increased excitability), and 12 (20%) structural or functional heart abnormalities: seven (58%) ventricular hypertrophy and others structural or functional heart abnormalities—patent foramen ovale, pulmonary artery stenosis, atrioventricular block and atrial, perimembranous ventricular, and muscular ventricular septal defects. Five newborns had anomalies: two cleft palate or lip, one anomalies of the ears, and two hydronephrosis. In four newborns respiratory distress was present, the etiology being transient tachypnea of the newborn in three cases, and meconium aspiration syndrome in one. The demographic and clinical characteristic of the group are presented in Table 1.

**Table 1:** Demographic and clinical characteristics of newborns in correlation with maternal diagnosis of gestational diabetes mellitus.

Characteristics of newborns	Mothers with GDM (*n* = 24)	Mothers without GDM (*n* = 37)	Total (*n* = 61)	*p*-value
Sex, male, *n* (%)	12 (50)	26 (70)	38 (62)	0.185
Birth weight (grams), mean (SD)	4,275 (455)	4,220 (261)	4,268 (347)	0.907
Birth length (centimeters), mean (SD)	54.5 (1.9)	54.3 (2.0)	54.4 (1.9)	0.635
Head circumference (centimeters), median (SD)	36 (1.1)	36 (1.1)	36 (1.1)	0.856
Gestational age (week), median (SD)	40 (1.4)	40 (1.3)	40 (1.4)	0.866
Mother's age (year), mean (SD)	31.9 (5.0)	32.2 (5.5)	32.0 (5.3)	0.821
Adverse perinatal outcome, *n* (%)	13 (54)	18 (49)	31 (51)	0.874
Hypoglycemia, *n* (%)	3 (12.5)	2 (5.4)	5 (8.2)	0.331
Polycythemia, *n* (%)	3 (12.5)	1 (2.7)	4 (6.6)	0.134
Hyperbilirubinemia, *n* (%)	4 (16.7)	7 (18.9)	11 (18.0)	0.822
Respiratory distress, *n* (%)	1 (4.2)	3 (8.1)	4 (6.6)	0.532
Structural or functional heart abnormality, *n* (%)	8 (33.3)	4 (10.8)	12 (19.7)	0.031*
Mildly abnormal neurological signs, *n* (%)	6 (25.0)	6 (16.2)	12 (19.7)	0.403
Birth trauma, *n* (%)	3 (12.5)	3 (8.3)	6 (10.0)	0.601
Anomalies, *n* (%)	3 (12.5)	2 (5.4)	5 (8.2)	0.331

GDM, gestational diabetes mellitus; SD, standard deviation.

* *p* < 0.05.

Twenty-four LGA newborns were born to mothers diagnosed with GDM. They more frequently had abnormal heart ultrasound findings, mostly ventricular hypertrophy; otherwise, they did not differ from the group of newborns born to mothers without GDM (Table 1).

### 3.2 Clinical characteristics of mothers of LGA newborns

GDM was diagnosed prenatally in 24 of 61 mothers. All the women included took the recommended medication during pregnancy, for example, folic acid, iron, or vitamins; and some used progesterone in early pregnancy, none of the participants used drugs or alcohol, except for one woman who smoked during pregnancy. The median HbA1c level was lower in women without GDM (Table 2). The majority (20; 83%) of women with GDM followed a diet to regulate glucose levels, and the remaining four received insulin therapy. In two pregnancies with diagnosis of GDM, antenatal polyhydramnios were observed; one of them was treated with insulin.

**Table 2:** Glycated hemoglobin A1c and glycated albumin levels in mothers of large-for-gestational-age newborns in correlation with gestational diabetes mellitus.

	Mothers with GDM (*n* = 24)	Mothers without GDM (*n* = 37)	Total (*n* = 61)	*p*-value
HbA1c (%), median (SD)	5.45 (0.51)	5.15 (0.48)	5.20 (0.50)	0.102
GlyA (%), median (SD)	16.1 (4.68)	16.9 (4.65)	16.4 (4.6)	0.516
Minimum–maximum	9.7–25.8	10.7–32.2		
GlyA (µmol/L), median (SD)	79.5 (22.8)	82.0 (22.1)	81.0 (22.2)	0.842
Minimum–maximum	55.0–130.0	54.0–150.0		

GDM, gestational diabetes mellitus; GlyA, glycated albumin; HbA1c, glycated hemoglobin A1c; SD, standard deviation.

### 3.3 GlyA in mothers of LGA newborns regarding the presence of GDM

**Figure 1:**
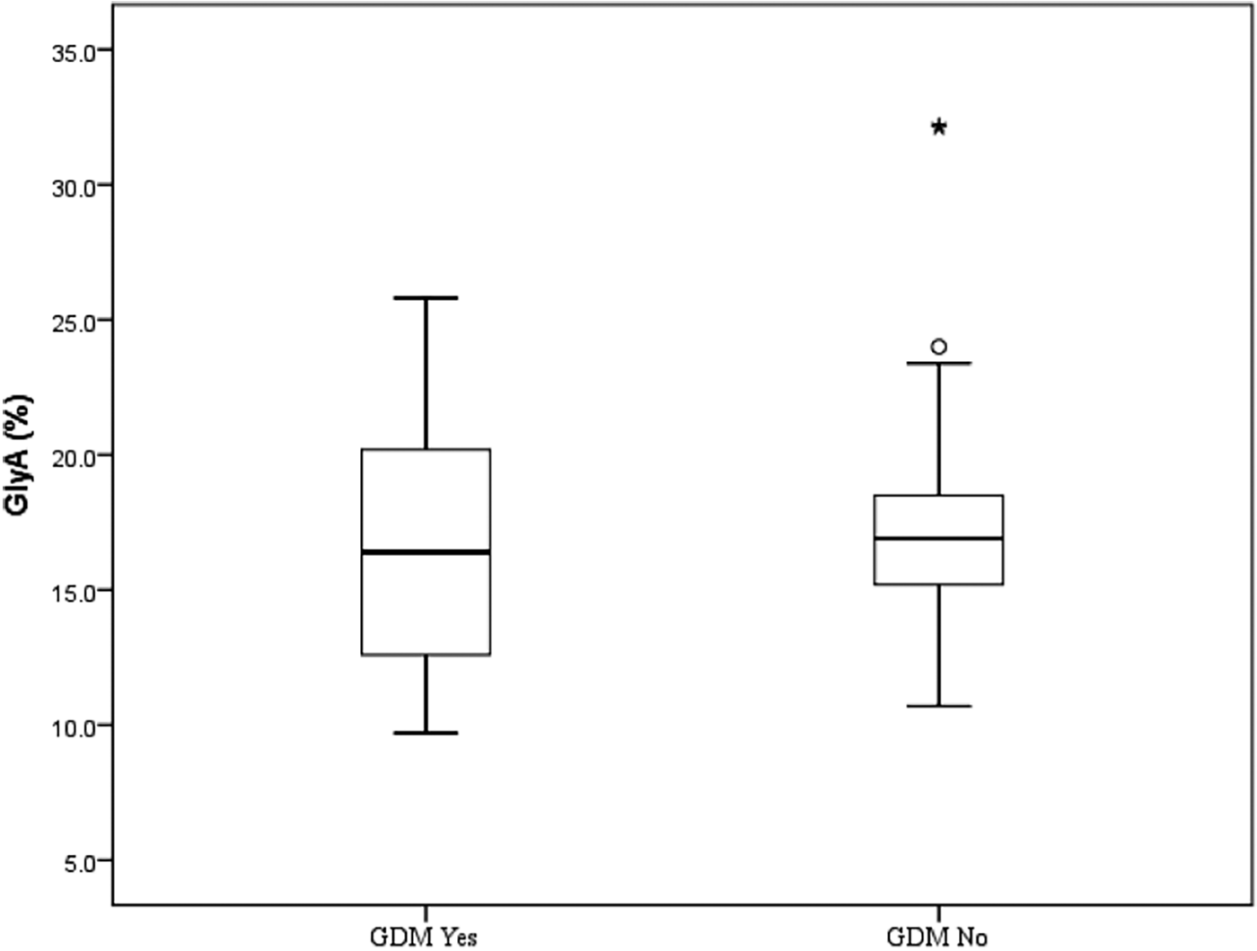
Glycated albumin (%) in mothers of large-for-gestational-age newborn in correlation with gestational diabetes mellitus. Each box represents the 25th to 75th percentiles. Lines inside the boxes ° represent the median. Lines outside the boxes represent the 10th and 90th percentiles. * Represents extreme value, represents potential outlier. GDM No, prenatal diagnosis of GDM not present; GDM Yes, prenatal diagnosis of GDM present; GlyA, glycated albumin.

The median value of GlyA was 16.4% (range 9.7%–32.2%) or 81.0 µmol/L (range 54.0–150 µmol/L). Although the median value of GlyA was higher in the group without a history of GDM, no significant difference between the groups was detected (*p* > 0.05) (Figure 1 and Table 2).

### 3.4 Maternal glycated albumin in predicting birth weight and other adverse perinatal outcomes

**Figure 2:**
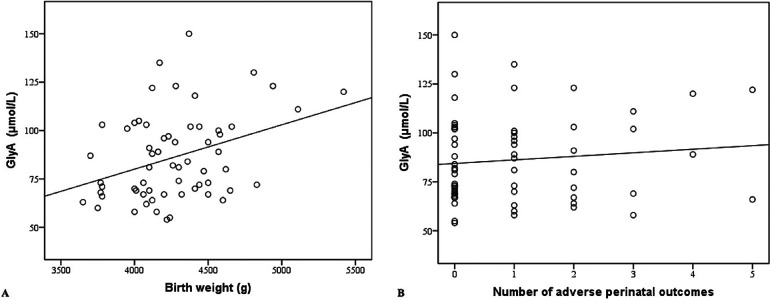
**(A)** Maternal glycated albumin in correlation with birth weight. **(B)** Maternal glycated albumin in correlation with number of adverse perinatal outcomes. GlyA, glycated albumin.

A multiple linear regression model was applied to determine the correlation between maternal GlyA levels, birth weight, and the number of adverse perinatal outcomes. We found that maternal GlyA levels were positively correlated with birth weight (*β* = 0.023, *p* = 0.007). If the number of adverse perinatal outcomes is constant, an increase in birth weight by an average of 1 g indicated an increase in the GlyA level at 0.023 µmol/L (Figure 2A). No correlations between the GlyA level and the number of adverse perinatal outcomes (*β* = 0.392, *p* = 0.855) were found (Figure 2B).

## 4 Discussion

To the best of our knowledge, this study is the first to determine postnatal GlyA levels in mothers after delivery of LGA newborns. We found that mothers of LGA newborns who were not diagnosed with GDM during pregnancy had higher median levels of GlyA and lower HbA1c levels than mothers with prenatal diagnosis of GDM, but these differences were not statistically significant. Values of GlyA in mothers were positively correlated with the birth weight of their newborns but no correlation with other adverse perinatal outcomes was found.

GlyA could potentially provide insight into glycemic control in the last trimester of pregnancy and thus influence the management of pregnant women. Several studies have shown that both HbA1c and GlyA can be used for screening GDM, including a recent meta-analysis revealing a significant correlation between elevated levels of GlyA and HbA1c and an increased risk of GDM (37). HbA1c levels are affected by iron deficiency anemia, whereas GlyA levels are not affected by erythrocyte survival, and may reflect blood glucose levels in past 2–3 weeks. GlyA correlates more strongly with fasting and postprandial blood glucose levels and glucose fluctuations and may be a more sensitive indicator of GDM (13, 19, 27, 38). Many authors show that episodic hyperglycemia, which is not reflected in HbA1c, is more likely to contribute to fetal growth acceleration than basal levels of blood glucose (38–41). Furthermore, GlyA is relatively easy to measure and appears to be a useful indicator of GDM (18, 28). It reflects intermediate-term glycemia, and changes rapidly and markedly, which could more accurately reflect glycemic status (18, 25, 28). In contrast, a study in pregnant women with Type 1 diabetes showed that HbA1c has better predictive power for obstetric and neonatal outcomes than alternative laboratory markers, including GlyA (42).

The reference interval of GlyA in non-pregnant Americans was reported to be 11.9%–15.8% (43), which is comparable to that in the Chinese population (27). Studies demonstrated that the level of GlyA decreased as pregnancy progressed (18, 44). A Japanese study showed that the reference range of GlyA in healthy Japanese pregnant women was between 11.5% and 15.7% (18). The value of GlyA in detecting adverse perinatal outcomes has also been investigated in several studies. Li et al. performed a study in Chinese women with GDM, and suggested that the risk of macrosomia increased significantly when the GlyA level was ≥13.0% and ≥12.0% at 24–28 and 26–38 weeks of gestation, respectively. They also found that the GlyA level decreased gradually in both normal and GDM pregnancies (25). In the study by Dong et al. the authors found no significant difference in GlyA levels between pregnant women with and without GDM, as well as the limited significance of GlyA in predicting LGA and other adverse perinatal outcomes (29). Similarly, Chinese authors found no significant difference in GlyA values between pregnant women with and without GDM, but they demonstrated that the frequency of LGA and caesarean section deliveries was significantly higher when GlyA levels exceeded 15.69% in the third trimester of pregnancy (26). Sugawara et al. reported that maternal GlyA levels were significantly higher in a group of LGA newborns, and considered that the cut-off values for GlyA during late pregnancy to predict complications ranged from 13.6% to 14.7% (28). Although we studied the levels of GlyA after delivery, our results could be compared with the results of the study in Portuguese pregnant women with GDM, in which the researchers showed that GlyA levels measured within 4 weeks before delivery was better in predicting adverse perinatal outcomes, particularly birth weight and LGA newborns, than HbA1c (45). Recently, Omokhodion and coauthors reported a good correlation between postnatal GlyA values in obese women and their newborns (46).

In our study, the median GlyA value in mothers of both groups of LGA newborns was higher than that in previously published studies of pregnant women with or without GDM (18, 25, 26, 45). We believe that this could be because we exclusively studied LGA newborns, and hypothesize that their accelerated growth could be caused by hyperglycemia during pregnancy. Although we did not find a significant difference in the level of GlyA between the two groups, the median value of GlyA was higher in the group without GDM. Considering this, and the fact that the HbA1c levels—as a blood glucose marker for the previous 2–3 months—were lower in women without prenatal diagnosis of GDM than in women with GDM, we may further hypothesize that the etiology of accelerated growth in our study was unrecognized and untreated GDM that developed after previous screening, in the third trimester of pregnancy. Moreover, our results show that the GlyA level is positively correlated with birth weight, indicating the importance of GlyA in predicting birth weight and adverse perinatal outcomes. Our hypothesis is also supported by the results of the study by Huidobro et al. in which the authors demonstrated that 35% of women with GDM developed GDM in the third trimester of pregnancy (47), as well as another prospective study in which the authors showed that out of 334 pregnant women, 13.5% had abnormal OGTT results in the third trimester after a negative test for GDM in the first and second trimesters (48). Recently, Guleroglu and coauthors demonstrated that GlyA or fetal pancreas size could be used for the early prediction of GDM (49). Additionally, some studies also suggest that women with a negative OGTT result in the second trimester may have glucose intolerance due to weight gain in late pregnancy (50). Several studies have also shown that the excessive fetal growth of LGA newborns begins in the second half of pregnancy and continues as pregnancy progresses (39, 40, 51), and that interventions initiated in the third trimester may reduce fetal growth (52).

A key strength of our study lies in examining GlyA levels in mothers of LGA newborns, thereby demonstrating valuable insight into GlyA as a potential biomarker for glycemic control in GDM. Despite this strength, our study has several limitations. First, GlyA levels were determined only once after delivery. We know from previous studies that GlyA levels decrease as pregnancy progresses, so we can assume that levels were higher during pregnancy. Second, we studied levels of GlyA and HbA1c only in a group of LGA newborns and did not compare them with values in the control group of appropriate for gestational age newborns according to the diagnosis of GDM in their mothers. Additionally, metabolic factors, such as weight gain, dietary patterns and maternal blood sugar values during pregnancy were not available for analysis. And, most importantly, the study was conducted at a single center and the sample was too small to detect significant differences between groups and to generalize our findings. We believe that our results should be validated in a large multicenter study to substantiate the present observations and to determine the utility and validity of GlyA as an alternative indicator of glycemic control in GDM and its diagnostic significance in predicting LGA newborns and their adverse perinatal outcomes. Finally, the reference intervals of GlyA at different gestational ages, in different ethnic communities and assay types, as well as cut-off values of GlyA, need further investigation.

In conclusion, we demonstrated that mothers of LGA newborns who were not diagnosed with GDM during pregnancy had higher median levels of GlyA and lower HbA1c levels than mothers with prenatal diagnosis of GDM, but these differences were not statistically significant. Values of GlyA in mothers were positively correlated with the birth weight of their newborns but no correlation with other adverse perinatal outcomes was found. Although additional larger studies are needed, our results indicate the potential value of GlyA for screening of GDM in the last trimester of pregnancy, especially as a biomarker associated with LGA newborns.

## Data Availability

The raw data supporting the conclusions of this article will be made available by the authors, without undue reservation.
